# Deviating from the Recommended Torque on Set Screws Can Reduce the Stability and Fatigue Life of Pedicle Screw Fixation Devices

**DOI:** 10.3390/medicina58060808

**Published:** 2022-06-15

**Authors:** Lien-Chen Wu, Yueh-Ying Hsieh, Fon-Yih Tsuang, Yueh-Feng Chiang, Chang-Jung Chiang

**Affiliations:** 1 Department of Orthopedics, Shuang Ho Hospital, Taipei Medical University, New Taipei City 23561, Taiwan; d98548019@tmu.edu.tw (L.-C.W.); 11154@s.tmu.edu.tw (Y.-Y.H.); 2 Department of Orthopaedics, School of Medicine, College of Medicine, Taipei Medical University, Taipei 110, Taiwan; 3 Graduate Institute of Biomedical Materials and Tissue Engineering, College of Bio-Medical Engineering, Taipei Medical University, Taipei 110, Taiwan; 4 Division of Neurosurgery, Department of Surgery, National Taiwan University Hospital, Taipei 100, Taiwan; tsuangfy@ntu.edu.tw; 5 Department of Orthopaedics, Taichung Tzu Chi Hospital, Taichung 427, Taiwan; cyf@tzuchi.com.tw

**Keywords:** pedicle screw, torque on set screw, fatigue life, gipping capacity, over-tightening

## Abstract

*Background and Objectives*: Using an appropriate torque to tighten set screws ensures the long-term stability of spinal posterior fixation devices. However, the recommended torque often varies between different devices and some devices do not state a recommended torque level. The purpose of this study is to evaluate the effect of set screw torque on the overall construct stability and fatigue life. *Materials and Methods*: Two commercial pedicle screw systems with different designs for the contact interface between the set screw and rod (Group A: plane contact, Group B: line contact) were assembled using torque wrenches provided with the devices to insert the set screws and tighten to the device specifications. The axial gipping capacity and dynamic mechanical stability of each bilateral construct were assessed in accordance with ASTM F1798 and ASTM F1717. *Results*: Increasing or decreasing the torque on the set screw by 1 Nm from the recommended level did not have a significant effect on the axial gripping capacity or fatigue strength of Group A (*p* > 0.05). For Group B, over-tightening the set screw by 1 Nm did cause a significant reduction in the fatigue strength. *Conclusions*: Excessive torque can damage the rod surface and cause premature failure. When insertion using a manual driver is preferred, a plane contact interface between the set screw and rod can reduce damage to the rod surface when the set screw is over-torqued.

## 1. Introduction

Pedicle screws are commonly used to treat disorders of the spine through instrumented spinal fusion. The aim is to correct deformities and stabilize the spine to safely achieve arthrodesis. Stiff and stable constructs have been shown to improve fusion rates and increase the strength of the fusion mass [1,2].

Spinal surgical techniques have evolved considerably over the past 10 years, particularly with the use of minimally invasive surgery where pedicle screws can now be inserted through a 2–3 cm incision [3]. Open surgery typically involves prolonged use of anesthetics, long operation time, prolonged hospital course, blood loss, and damage to neural/soft tissues. While a minimally invasive approach can overcome many of these disadvantages of open surgery, the procedure often requires the surgeon to use their hands to manually control set screw insertion. The use of power and precision instrumentation is limited by such a small incision window. Hence, surgical errors, such as an inappropriate insertion torque on the set screw, are more common than with the open approach [4].

Standard top-loading pedicle screws have a tulip-type screw head with a U-shaped slot to accommodate the rod, which is held in place by a set screw. The clamping force of the system is mainly determined by the ability of the set screw to maintain a tight clamp on the rod when the rod is subjected to a radial force. The set screw can be torqued manually using screwdrivers, but this is prone to error with variation in the torque applied. Inadequate torque may lead to set screw loosening and failure, while excessive torque may lead to fracture of the rod [5,6,7]. Therefore, it is essential to accurately torque each set screw within the range recommended by the manufacturer. Studies on dental implants have shown that most dentists cannot accurately torque abutment screws using manual drivers [8,9], and there is a wide variation between operators [10]. We propose that there is a similar variation in the ability of spinal surgeons to perceive adequate torque when inserting set screws during internal spinal fixation.

Implant disassembly due to loosening the set screw is uncommon, but insufficient tightening of the inner nut into the pedicle screw head can predispose the system to rod migration [6,11]. Few studies have investigated this failure mode, and most are in the form of case reports [5,6,7]. In contrast, excessive torque may affect the fatigue cycle of an implant. Therefore, the degree of tightening of the set screw plays an important role in structural stabilization.

Some rod and screw systems are supplied with a torque-limiting driver or breakaway set screw design to allow for consistent tightening of all set screws. However, this is not standard with all pedicle screw systems, and the recommended driving torque is often not detailed in the instructions supplied with the product. The effect of set screw torque on construct stability and screw loosening has not been investigated to date. The aim of this study is to evaluate the stability of commercially available pedicle screw constructs tested according to ASTM F1798 and ASTM F1717 using different torques for the set screws. Insufficient preloading or inadequate tightening of set screws can create stress concentrations and compromise implant stability. Hence, this study will investigate the relationship between the tightening torque and overall construct stability to improve understanding for clinical applications.

## 2. Materials and Methods

**Figure 1:**
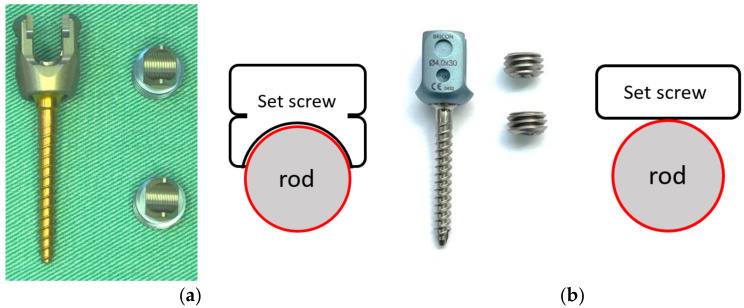
(**a**) MATRIX Spine System; plane contact surface between set screw and rod. (**b**) OCTOPODA Spine System; line contact surface between set screw and rod.

Two commercially available pedicle screw and rod systems were assessed in this study, both using 4.0 mm-diameter screws that accommodate a 5.5 mm-diameter rod (Table 1, Figure 1). Both systems are CE-marked, meeting the standards of equivalency with existing devices. The set screws used to secure the pedicle screws to the spinal rods were tightened according to specification using a torque wrench provided with each device. Bilateral constructs were tested for axial gipping capacity and dynamic mechanical loading in accordance with ASTM F1798 [12] and ASTM F1717 [13], respectively, using an MTS MiniBionix testing system (MTS Systems Corporation, Eden Prairie, MN, USA) with an MTS axial/torsional load cell (model 662.20H-05). The axial and torsional capabilities were 25 KN and 250 Nm, respectively.

### 2.1. Axial Gipping Capacity (AGC) Testing

**Figure 2:**
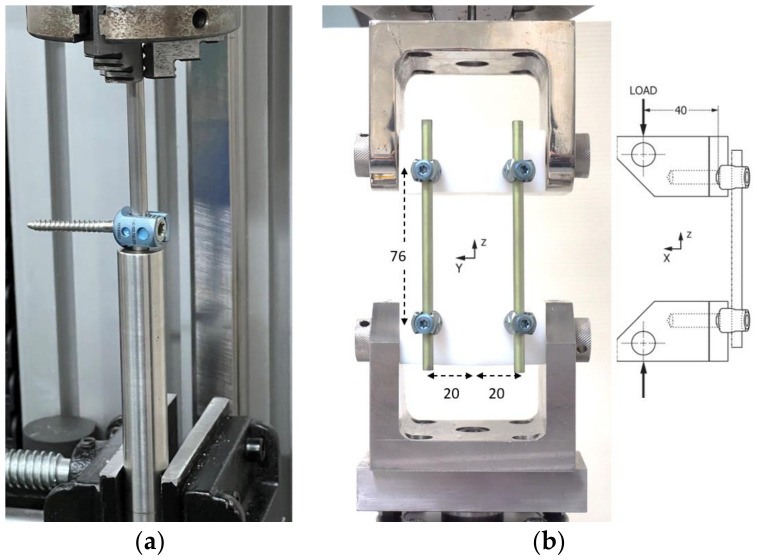
(**a**) Setup of axial gripping capacity (AGC) testing. (**b**) Setup of dynamic mechanical testing.

**Figure 3:**
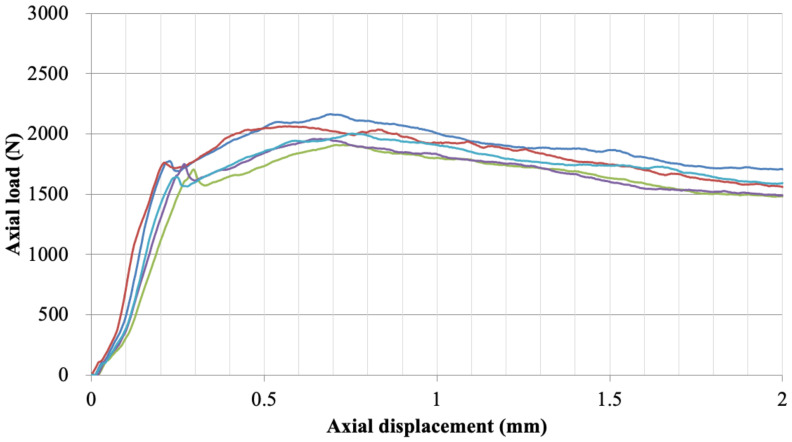
Sample testing curve for axial slip testing showing five test runs for a group.

The axial gripping capacity (AGC) was assessed using 15 pedicle screws and 15 spinal rods for each device (Group A and B). One end of the spinal rod was clamped to the actuator using a collet. A poly-axial screw assembly was then placed on the load cell and rested on a metal tube with a recess to allow the rod to advance (Figure 2a). A pedicle screw was secured to the rod 20 mm from the collet end with 5 mm of rod left beyond the interconnection. Pedicle screw heads that were not flush with the test machine base were supported with a washer to evenly distribute the load around the interconnection. Axial loading was applied at a rate of 15 mm/min until 5 mm of displacement was achieved. The axial gripping capacity (AGC) of each construct was defined as the maximum load (N) supported within the initial 1.5 mm of displacement [14] (Figure 3). Set screws were tightened at three different torque values to simulate standard insertion (manufacturer recommended), over-tightening and under-tightening. Five samples were tested at each torque value. A student’s *t*-test was used to detect significant differences in AGC for different torque values (*p* < 0.05 indicates a statistically significant difference).

### 2.2. Dynamic Mechanical Testing of Bilateral Constructs

Dynamic mechanical testing was performed on 11 bilateral constructs of each device, in total consisting of 44 pedicle screws and 22 rods. Blocks of ultra-high molecular weight polyethylene (UHMWPE) were formed according to ASTM F1717. As shown in Figure 2b, two screws were secured in each block and linked by rods to simulate fixation between two adjacent vertebrae. The constructs (rods and screws) were set up according to the manufacturer’s instructions using standard surgical instruments. The active length was 76 mm, and the moment arm from the centerline to the insertion point was 40 mm on the *x*-axis and 20 mm on the *y*-axis. Set screws were tightened at three different torque values to simulate standard insertion (manufacturer recommended), over-tightening by 1 Nm and under-tightening by 1 Nm. Dynamic compression bending tests were performed on both constructs according to the methods described in ASTM F1717 [13]. All tests were conducted in dry air at room temperature. For the dynamic fatigue test, the frequency was set at 5 Hz with a cyclic sine wave. The R value (minimum load divided by maximum load) was 10. The test was ceased when the sample failed (meaning any permanent deformation that altered the functional performance), the distance between the test blocks was reduced to less than 38 mm, or when 5,000,000 cycles (run-out) were reached. The load applied and the duration of loading were recorded to determine the fatigue strength. ASTM F1717 recommends that loading commences at 50% of the ultimate load, which in this study was determined to be 340 N and 355 N for Group A and B by using a preliminary static test. Therefore, loading started at 170 N for both groups and was increased after every third sample until permanent deformation or functional failure occurred, or the number of cycles exceeded 5,000,000 cycles. Otherwise, the load level was decreased every third sample until sample run-out. The fatigue strength for both Group A and B was 190 N when torquing the set screw to the manufacturer’s recommended specification.

## 3. Results

### 3.1. Axial Gripping Capacity

Group A had an average AGC of 2019.65 N when using the manufacturer’s recommended torque to tighten the set screw, while the AGC increased or decreased, respectively, when the torque value was higher or lower than the standard insertion (Table 2). There was no significant difference (*p* > 0.05) between the AGC values recorded for Group A. Group B had an average AGC of 1775.07 N using the standard insertion toque, and over-tightening the set screw did not significantly affect the gripping capacity (*p* > 0.05). However, there was a significant decrease in AGC when the set screw was under-tightened (*p* < 0.05).

### 3.2. Dynamic Mechanical Testing of Bilateral Construct

**Figure 4:**
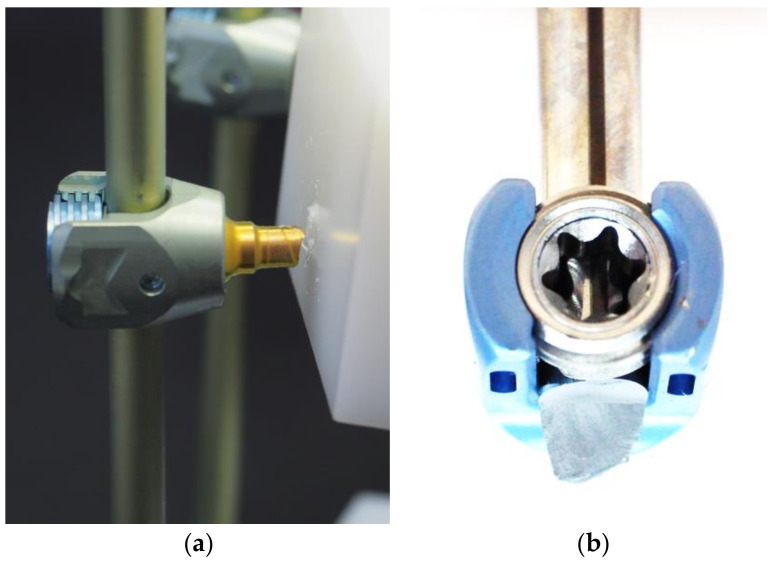
Failure patterns from the dynamic compression bending test in (**a**) Group A and (**b**) Group B.

Table 3 and Table 4 details the results of the dynamic compression bending test. All constructs in Group A had a fatigue strength of 190 N regardless of the torque used to tighten the set screw. Where the construct failed within 5,000,000 cycles, failure was through fracture of the pedicle screw at the point where it entered the UHMWPE block (Figure 4). Group B had a fatigue strength of 190 N when using the manufacturer’s recommended torque (12 Nm). Decreasing the torque had no discernible effect, but increasing it to 13 Nm did lead to a reduction in fatigue strength to 170 N (Table 3). In cases where 5,000,000 cycles were not reached, the construct failed by fracture of the rod.

## 4. Discussion

Internal fixation with top-loading pedicle screw systems requires the set screws to be sufficiently tightened to hold the rod in place and achieve a secure fixation and symmetric load condition. It has been reported that operators using hand-held manual drivers can show considerable variation in the torque applied [10,15]. The ability to accurately torque a set screw is essential for long-term implant stability. Through clinical experience, the authors are aware of cases of postoperative set screw loosening, and studies have shown that the insertion torque is a critical factor [5,6,7,16,17]. Hence, this study evaluated the mechanical performance of posterior fixation systems incorporating pedicle screws and rods using different tightening torques for the set screws. The hypothesis was that deviating from the recommended torque on the set screws would damage the construct stability.

The function of set screws is to secure the rod to the poly-axial mechanism. Previous reports indicated that inadequate tightening of the set screws could result in rod disengagement from the screw head [18]. In the axial gripping capacity test, for both constructs tested, the holding load between the set screw and rod increased with the torque. A mechanical study involving three custom-made pedicle screws by Liu et al. [19] showed that the gripping capacity of a poly-axial screw using a 12 Nm tightening torque was significantly greater than with an 8 Nm tightening torque. This finding is similar to the results of our study, indicating that a greater torque could reduce the risk of rod slippage. Although the OCTOPODA construct (Group B) used a greater tightening torque than Group A, the average axial gripping strength of Group B was lower. The design of the set screw may be the main reason for the different grip strengths. The saddle on the underside of the set screw in Group A has a groove and ridge geometry to increase the holding area of the rod on the screw head, whereas Group B had a line contact with a smaller profile (Figure 1) [20]. Liu et al. [19] indicated that gaps between the tulip-rod interface, such as with the OCTOPODA implant (Group B), can lead to a reduction in holding force. 

**Figure 5:**
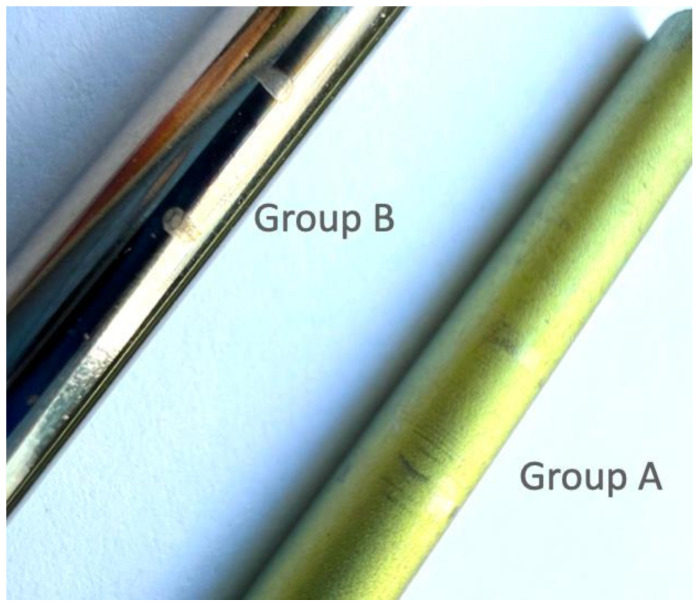
The contact surface between the set screw and Ti6Al4V rod in Groups A and B after tightening the tulip head of the pedicle screw.

Even in cases where a torque-limiting driver is supplied with the implant, an orthopedic surgeon still has the option to tighten the set screw manually. Loosening set screws after implantation has been reported with an incidence rate of 2–12% at the long-term follow up [21,22]. To mitigate the known potential for screw loosening, some surgeons over-tighten the set screws. However, the results of the compression bending test in this study showed a considerable reduction in fatigue strength of the construct when tightening the screw beyond the recommended torque. All samples in Group A had a fatigue strength of 190 N, with the primary failure mode being fracture around the proximal neck of the screw where it enters the test block. There was a noticeable reduction in fatigue strength in Group B as the insertion torque on the set screw was increased to 13 Nm, and all implants that did not achieve run-out failed by fracture of the rod at the region where it connects with the pedicle screw. We considered that excessive torque on the set screw could damage the contact surface of the rod, which would reduce the fatigue strength. The low contact profile between the rod and screw in Group B, defined as ‘line contact’ in this study, may produce localized stress concentrations that would exceed the plane contact stresses experienced by Group A. Figure 5 shows the depression on the rod in Group B at the region where it contacts the set screw, whereas only minor scratch marks were observed in Group A. This may explain the difference in failure modes. In a retrieval study on PEEK rods, Kurtz et al. [23] predominantly observed plastic deformation of the rods caused by indentations by set screws. Hence, the recommended torque for tightening set screws when used in conjunction with PEEK rods is usually lower than with metal rods. To reduce the incidence of pedicle screw failure, it is recommended that surgeons use a torque-limiting driver adjusted to the manufacturer’s recommended torque.

This study has some limitations. First, only two commercially available poly-axial screws were evaluated using three torque values for the set screws. The methods used are adequate for this study because the intent was to investigate how over- and under-tightening of set screws affects the performance of the construct. In addition, testing was performed in accordance with ASTM F1798 and ASTM F1717 rather than aiming to replicate physiological conditions, so rod bending and variations in the poly-axial tilt angle were not evaluated. ASTM standards are typically used to compare different component designs or surgical techniques in terms of relative mechanical parameters [24,25]. Finally, the mechanical properties of the two rods were not evaluated in this study. However, a previous investigation by the authors using four-point bending on the rods in Groups A and B showed that the rods have similar mechanical properties; please refer to the Supplementary Materials (Supplemental Reports S1). Any difference in properties between the rods would have a negligible effect on the test results.

## 5. Conclusions

Within the limitations of this study, the results demonstrated the importance of achieving the target torque values when tightening the set screw. Insufficient torque can lead to rod slippage or failure of the poly mechanism, while excessive torque can damage the rod surface and reduce the fatigue life. A line contact surface between the set screw and rod can damage the rod surface when the screw torque exceeds the recommended level. The optimal torque range for the set screw often depends on the design and properties of the construct, and, as such, there is no universal torque suitable for all devices. The authors recommend that the target torque range be stated with all devices, and a torque-limiting driver be provided as standard instrumentation for non-breakaway set screw systems. In pre-surgery planning, surgeons should confirm the torque limit handles with manufacturer-recommended torque or set screws with the breakaway head feature have been provided in instrument and implant sets, respectively.

## Figures and Tables

**Table 1:** Specifications of implants assessed in this study.

Pedicle Screw	A (Matrix) *	B (OCTOPODA) **
Rod material	Ti alloy	Ti alloy
Screw diameter (mm)	4.0	4.0
Screw length (mm)	30	30
Screw material	Ti alloy	Ti alloy
Set screw material	Ti alloy	Ti alloy
Tightening torque (Nm)	9 (lower) 10 (Manufacturer recommended) 11 (higher)	11 (lower) 12 (Manufacturer recommended) 13 (higher)

* MATRIX, DePuy Synthes, Oberdorf, Switzerland. ** OCTOPODA, Bricon GmbH, Wurmlingen, Germany.

**Table 2:** Comparison of axial gripping capacity (AGC) between test groups.

Group (Torque Value of Set Screw)	Mean	SD *	*p* Value **
A (9 Nm)	1940.23	102.10	0.123 compared to A (10 Nm)
A (10 Nm)	2019.65	98.81	-
A (11 Nm)	2067.30	105.66	0.241 compared to A (10 Nm)
B (11 Nm)	1651.23	88.05	0.031
B (12 Nm)	1775.70	94.78	-
B (13 Nm)	1818.97	69.25	0.216

* SD standard deviation. ** Bold values indicate statistical significance with a *p* value < 0.05.

**Table 3:** Results of the dynamic compression bending test.

Sample No. Group A	Torque Value of Set Screw (Nm)	Range of Axial Force (N)	Cycles to Failure
1	10	22~220	53,548
2	10	20~200	2,177,963
3	10	20~200	1,757,238
4	10	19~190	Run-out
5	10	19~190	Run-out
6	9	19~190	Run-out
7	9	19~190	Run-out
8	11	19~190	Run-out
9	11	19~190	Run-out
10	11	20~200	1,831,177
11	11	20~200	1,593,474

**Table 4:** Results of the dynamic compression bending test for two test groups.

Group (Torque Value of Set Screw)	* Fatigue Strength (N)	Maximum Axial Force (N)	Failure Patten If Did Not Run-Out
A (9 Nm)	190	22~220	Screw fracture
A (10 Nm)			
A (11 Nm)			
B (11 Nm)	190	22~220	Rod fracture
B (12 Nm)			
B (13 Nm)	170	19~190	

* Run-out: run out at 5 million cycles as recommended by ASTM F1717.

## Data Availability

All relevant data are within the manuscript.

## Supplementary Materials

The following supporting information can be downloaded at: https://www.mdpi.com/article/10.3390/medicina58060808/s1, Report S1: ASTM F2193-20:A3 Specification for metallic spinal rods.

## References

[1] Groff M.W., Dailey A.T., Ghogawala Z., Resnick D.K., Watters W.C., Mummaneni P.v., Choudhri T.F., Eck J.C., Sharan A., Wang J.C. (2014). Guideline update for the performance of fusion procedures for degenerative disease of the lumbar spine. Part 12: Pedicle screw fixation as an adjunct to posterolateral fusion. J. Neurosurg. Spine.

[2] Boos N., Webb J.K. (1997). Pedicle screw fixation in spinal disorders: A European view. Eur. Spine J..

[3] Landriel F., Guiroy A., Ciancio A.M., Taboada N., Menezes C., Gotfryd A., Kornfeld S., Hem S. (2021). 20 tips to avoid and handle problems in the placement of percutaneous pedicle screws. World Neurosurg..

[4] Lykissas M.G., Giannoulis D. (2018). Minimally invasive spine surgery for degenerative spine disease and deformity correction: A literature review. Ann. Transl. Med..

[5] Kumar P., Kumar V., John R., Sharma R. (2017). Early loosening of spinal rod in a case of degenerative grade 1 spondylolisthesis treated with unilateral pedicle screw fixation and transforaminal cage for interbody fusion. J. Orthop. Case Rep..

[6] Agarwal A. (2014). Improper coupling between inner nut and screw head leading to rod loosening and dislodgement. Rom. Neurosurg..

[7] Krishnan P., Das S. (2021). Implant disassembly due to pedicle screw nut loosening. J. Neurosci. Rural. Pract..

[8] Vallee M.C., Conrad H.J., Basu S., Seong W.J. (2008). Accuracy of friction-style and spring-style mechanical torque limiting devices for dental implants. J. Prosthet. Dent..

[9] Jaarda M.J., Razzoog M.E., Gratton D.G. (1994). Effect of preload torque on the ultimate tensile strength of implant prosthetic retaining screws. Implant. Dent..

[10] Neiburger E.J. (1994). Dentists’ age and manual dexterity. Are younger or older dentists better practitioners?. Gen. Dent..

[11] Serhan H., Hammerberg K., O’Neil M., Sturm P., Mardjetko S., Crawford A. (2010). Intraoperative techniques to reduce the potential of set-screw loosening in long spinal constructs: A static and fatigue biomechanical investigation. J. Spinal. Disord. Tech..

[12] Standard Test Method for Evaluating the Static and Fatigue Properties of Interconnection Mechanisms and Subassemblies Used in Spinal Arthrodesis Implants. https://www.astm.org/f1798-21.html.

[13] Standard Test Methods for Spinal Implant Constructs in a Vertebrectomy Model. https://www.astm.org/f1717-21.html.

[14] Kluck D.G., Farnsworth C.L., Jeffords M.E., Marino N.E., Yaszay B., Upasani V.v., Newton P.O. (2020). Spinal rod gripping capacity: How do 5.5/6.0-mm dual-diameter screws compare?. Spine Deform..

[15] Al-Otaibi H.N. (2016). Intended and achieved torque of implant abutment’s screw using manual wrenches in simulated clinical setting. J. Contemp. Dent. Pract..

[16] Zdeblick T.A., Kunz D.N., Cooke M.E., McCabe R. (1993). Pedicle screw pullout strength. correlation with insertional torque. Spine.

[17] Okuyama K., Abe E., Suzuki T., Tamura Y., Chiba M., Sato K. (2000). Can insertional torque predict screw loosening and related failures? An in vivo study of pedicle screw fixation augmenting posterior lumbar interbody fusion. Spine.

[18] Voleti P.B., Shen F.H., Arlet V. (2014). Failure of monoaxial pedicle screws at the distal end of scoliosis constructs: A case series. Spine Deform..

[19] Liu P.Y., Lai P.L., Lin C.L. (2019). A Biomechanical investigation of the retentive force of pedicle screw structures for different screw tulip designs. Clin. Biomech..

[20] Ardura F., Chenaux D., Pascal-Moussellard H., Hessmann M.H. (2021). Evaluation of the reduction, tightening and gripping performance of an innovative set screw technology for instrumented posterior lumbar fusion: A biomechanical study. Orthop. Traumatol. Surg. Res..

[21] Jutte P.C., Castelein R.M. (2002). Complications of pedicle screws in lumbar and lumbosacral fusions in 105 consecutive primary operations. Eur. Spine J..

[22] Esses S.I., Sachs B.L., Dreyzin V. (1993). Complications associated with the technique of pedicle screw fixation. A selected survey of ABS members. Spine.

[23] Kurtz S.M., Lanman T.H., Higgs G., MacDonald D.W., Berven S.H., Isaza J.E., Phillips E., Steinbeck M.J. (2013). Retrieval analysis of PEEK rods for posterior fusion and motion preservation. Eur. Spine J..

[24] Yang T., Chen K., Lv Y., Yang T., Chen K., Lv Y. (2013). Fatigue life analysis of fixed structure of posterior thoracolumbar pedicle screw. Engineering.

[25] La Barbera L., Galbusera F., Wilke H.J., Villa T. (2016). Preclinical evaluation of posterior spine stabilization devices: Can the current standards represent basic everyday life activities?. Eur. Spine J..

