# Supplementary material for: Deviating from the Recommended Torque on Set Screws Can Reduce the Stability and Fatigue Life of Pedicle Screw Fixation Devices
Source: Medicina (Kaunas). 2022 Jun 15;58(6):808. doi: 10.3390/medicina58060808 (PMC9228452; doi:10.3390/medicina58060808)
Supplement: Supplementary file 1 [file medicina-58-00808-s001.zip › medicina-1745136-supplementary.pdf]

Supplement:

Purpose: For the spinal rods in Table 1, the purpose of this investigation is to determine the bending structural stiffness, bending yield moment, bending ultimate moment, bending fatigue runout moment, and when applicable, the median bending fatigue moment at 2,500,000 cycles.

Methods: Refer to the static and fatigue test methods from ASTM F2193-20:A3 "Specification for metallic spinal rods".

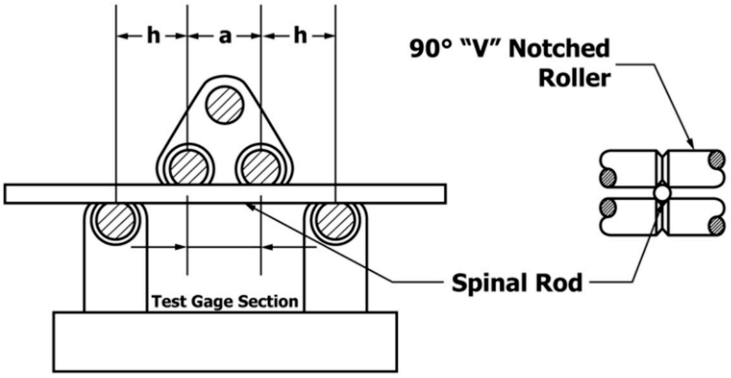

FIG. A3.1 Spinal Rod Test Configuration

Samples:

Table S1 Specifications of rods assessed in this test

Pedicle screw	**A (Matrix)***	**B (OCTOPODA)****
Rod material	Ti alloy	Ti alloy
Rod diameter (mm)	5.5	5.5
Rod length (mm)	120	120
Rod material	Ti alloy	Ti alloy

* MATRIX, DePuy Synthes, Oberdorf, Switzerland

** OCTOPODA, Bricon GmbH, Wurmlingen, Germany

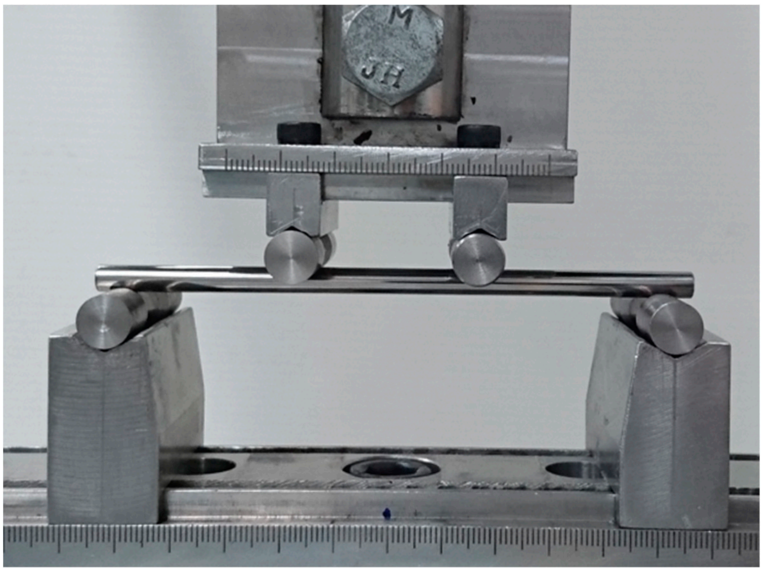

Figure S1. Test configuration.

Results:

Table S2 Results of static test

	Sample number (pcs)	Bending structural stiffness, N/mm² (Average, STD)	Bending yield moment, Nm (Average, STD)	Bending ultimate moment, Nm (Average, STD)
**Sample A**	5	4.20±0.15	20.42±0.52	26.16±0.19
**Sample B**	5	4.21±0.23	20.65±0.89	26.50±0.31

Table S3a Results of bending fatigue runout moment in group A

Axial load (N) / bending moment (N-m)	Sample (pcs)	Results (cycles)
**1275 / 19.125**	1	Rod failure (24,752)
**850 / 12.75**	1	Rod failure (1,512,317)
**680 / 10.20**	1	Run-out (>2,500,000)
**765 / 11.475**	1	Run-out (>2,500,000)
**850 / 12.75**	1	Rod failure (1,474,682)
**765 / 11.475**	1	Run-out (>2,500,000)

* bending fatigue runout moment: 765 N

Table S3b Results of bending fatigue runout moment in group B

Axial load **(N)** / bending moment (**N-m)**	Sample (pcs)	Results **(**cycles
**1275 / 19.125**	1	Rod failure (26,478)
**850 / 12.75**	1	Rod failure (1,477,693)
**680 / 10.20**	1	Run-out (>2,500,000)
**765 / 11.475**	1	Run-out (>2,500,000)
**850 / 12.75**	1	Rod failure (1,457,144)
**765 / 11.475**	1	Run-out (>2,500,000)

* bending fatigue runout moment: 765 N

Conclusion: Group A and B demonstrated a similar static mechanical performance with the same bending fatigue runout moment. These results show the mechanical properties of the two spinal rods are equivalent.
